# Anti-CD3 monoclonal antibody as a possible therapeutic alternative in HAM/TSP

**DOI:** 10.1186/1742-4690-11-S1-O40

**Published:** 2014-01-07

**Authors:** Johan Van Weyenbergh, Daniele Decanine, Saul Velloso Schnitman, Ana Maria Moro, Jorge Kalil, Ramon A Kruschewsky, Bernardo Galvão-Castro

**Affiliations:** 1 LIMI, LASP, Gonçalo Moniz Research Center (CPqGM), Oswaldo Cruz Foundation (FIOCRUZ), Salvador-Bahia, Brazil; 2 Institute for Immunological Investigation (iii-INCT), São Paulo, Brazil; 3 Bahiana School of Medicine and Public Health, Salvador-Bahia, Brazil

## 

Anti-CD3 antibody therapy is used to treat transplant rejection and has been applied in several autoimmune diseases, such as type I diabetes and ulcerative colitis, but its potential in HAM/TSP has not been investigated. Therefore, we explored the effect of ex vivo treatment (24-96h) of PBMCs from HAM/TSP patients (n=15) and healthy controls (n=20, all from Salvador-Bahia, Brazil) with an anti-CD3 monoclonal antibody approved for in vivo treatment post-transplantation (produced under GMP conditions). In contrast to normal donors and patients in early disease stages, anti-CD3 treatment did not increase lymphoproliferation in PBMCs from advanced HAM/TSP patients (EDSS>4), but strongly induced apoptosis. In addition, anti-CD3 treatment did not induce a pro-inflammatory cytokine storm, either at the protein or mRNA level. Therefore, anti-CD3 treatment ex vivo might eliminate pathogenic T cells through activation-induced cell death. Since we have previously shown that pro-apoptotic capacity decreases over time in HAM/TSP patients, both defective (Fas-mediated) apoptosis and excessive lymphoproliferation, more pronounced in patients with advanced disease progression, can be restored by anti-CD3 treatment. Using microarray analysis, we found that treatment of HAM/TSP PBMCs with anti-CD3 mAb had a pronounced effect on gene expression, significantly (p<0.001) down-regulating 1918 genes (including pro-inflammatory genes) and up-regulating 1926 genes, including cell cycle-related and immunoregulatory genes, such as CTLA4. In conclusion, our results suggest anti-CD3 monoclonal antibody might be a possible therapeutic alternative in HAM/TSP, due to its immunomodulatory effect, by inducing apoptosis, reducing exacerbated lymphoproliferation and stimulating anti-inflammatory gene expression and/or proliferation of cells with immunoregulatory capacity.

